# The Importance of Vaccinating Children and Pregnant Women against Influenza Virus Infection

**DOI:** 10.3390/pathogens8040265

**Published:** 2019-11-26

**Authors:** Ravi S Misra, Jennifer L Nayak

**Affiliations:** 1 Department of Pediatrics Division of Neonatology, The University of Rochester Medical Center, Rochester, NY 14623, USA; 2 Department of Pediatrics Division of Pediatric Infectious Diseases, The University of Rochester Medical Center, Rochester, NY 14623, USA; jennifer_nayak@urmc.rochester.edu

**Keywords:** pediatrics, influenza virus, vaccines, pregnant women, ARDS, lung, morbidity, vaccination rate

## Abstract

Influenza virus infection is responsible for significant morbidity and mortality in the pediatric and pregnant women populations, with deaths frequently caused by severe influenza-associated lower respiratory tract infection and acute respiratory distress syndrome (ARDS). An appropriate immune response requires controlling the viral infection through activation of antiviral defenses, which involves cells of the lung and immune system. High levels of viral infection or high levels of inflammation in the lower airways can contribute to ARDS. Pregnant women and young children, especially those born prematurely, may develop serious complications if infected with influenza virus. Vaccination against influenza virus will lead to lower infection rates and fewer complications, even if the vaccine is poorly matched to circulating viral strains. Maternal vaccination offers infants protection via antibody transmission through the placenta and breast milk. Despite the health benefits of the influenza vaccine, vaccination rates around the world remain well below targets. Trust in the use of vaccines among the public must be restored in order to increase vaccination rates and decrease the public health burden of influenza.

## 1. Introduction

Influenza virus infection is a major cause of morbidity and mortality around the world, with over 3 million individuals developing severe disease and resulting in hundreds of thousands of deaths per year [1]. Some of the most vulnerable populations include pregnant women and young children, making these groups a high priority target for vaccination. Individuals who are less than 21 years old are considered to be in the pediatric group and subsets include neonates (0 to 28 days), infants (29 days to 2 years), children (2 years to less than 12 years), and adolescents (12 years to 21 years) [2]. For individuals who are less than 5 years of age, pediatric influenza-associated infections are estimated at 90 million cases per year, with 1 million cases of influenza-associated severe acute lower respiratory tract infection and 28,000–111,500 deaths, the majority of which occur in developing countries [3]. Children less than a year of age are particularly susceptible to infection as they have little pre-existing immunity and may be too young to be vaccinated, relying upon transferred maternal immunity to protect against infection [4,5,6,7]. 

Several studies report that in fatal cases of influenza virus infection, increased inflammation, and virus are found in the alveoli [8,9,10,11,12,13]. Disruption of the alveolar region due to viral infection and increased inflammation can contribute to the development of acute respiratory distress syndrome (ARDS), which is a major health concern for children and pregnant women [14,15,16,17]. Vaccination can help prevent infection, which will, in turn, prevent acute lower respiratory tract infections and ARDS. Vaccines are either in the form of Inactivated Influenza Vaccine (IIV) or Live Attenuated Influenza Virus (LAIV) [18]. Current influenza vaccines target predominately the variable region of the hemagglutinin protein. This can allow for viruses to escape the immune system via mutation, leading to vaccine mismatch and increased viral spread [19,20,21,22,23,24,25,26,27]. Scientists are continuing work to develop a universal influenza vaccine that targets a less variable region of the influenza virus and is thus protective against a greater breadth of viral strains but, while substantial progress has been made, challenges remain [28,29,30]. This review will discuss our current understanding of the immune response to influenza vaccination, with a focus on the benefits of vaccinating pregnant women and children against influenza virus.

## 2. Body

### 2.1. Background

**Figure 1:**
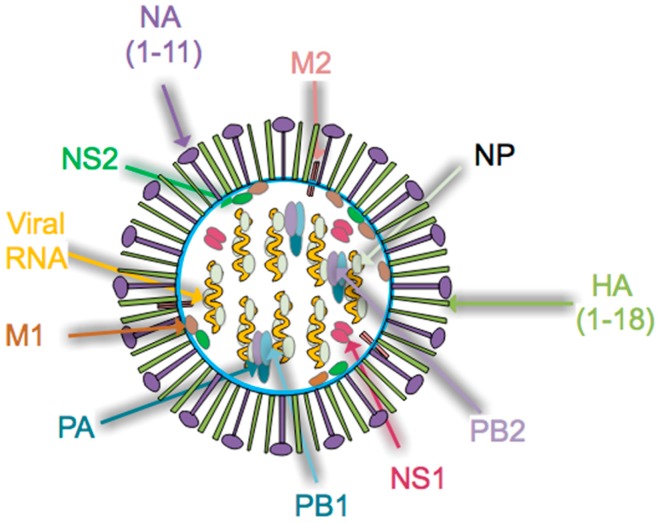
Diagram of the Orthomyxoviridae influenza virus. The virus consists of neuraminidase (NA), matrix protein 1 (M1), matrix 2 proton pump (M2), hemagglutinin (HA), polymerase acid subunits (PB1, PB2, PA), non-structural (NS1, NS2) and nucleoprotein (NP). Current vaccine strategies target the head regions of HA, which are highly variable between viral strains. Universal influenza vaccines target less-variable regions of the virus, such as the HA stalk, which will provide coverage for a range of influenza virus strains.

Influenza virus is a single stranded, negative sense Orthomyxoviridae RNA virus with a genome that contains 8 genomic segments [31]. Influenza A and B viruses include the hemagglutinin (HA), neuraminidase (NA), nucleoprotein (NP), matrix protein 1 (M1), matrix protein 2 (M2/BM2), nonstructural protein 1 (NS1), nonstructural protein 2 (NS2) and the RNA polymerase complex (PA, PB1 and PB2), as illustrated in Figure 1. The strain of influenza A virus is defined by the combination of HA and NA proteins, of which there are 18 and 11 distinct subtypes identified, respectively [31]. HA subtypes are classified as group 1 (H1, H2, H5, H6, H8, H9, H11, H12, H13, H16, H17, and H18) or group 2 (H3, H4 H7, H10, H14 and H15) [28]. While influenza A virus remains the major focus of public health officials, the pathogenic potential risk of influenza B virus infections must not be ignored [32,33]. In fact, several studies implicate influenza B viral infection as a substantial health concern in the young pediatric population [34,35,36]. Both molecular and host determinants, such as increased viral replication, host cell death, host antiviral gene response, degree of pre-existing immunity, and transmissibility, can contribute to the pathogenicity of influenza virus [37]. 

Given the relatively error-prone nature of the influenza virus RNA polymerases, mutations are often introduced which can result in viral escape from host immunity through the process of antigenic drift. Such mutations often are selected for in HA as they can lead to an escape from viral neutralization [19,20,21,22,23]. However, antigenic drift can also be seen in other viral proteins [24,25,27,38]. Alternatively, antigenic shift can occur when viral gene segment recombination results in formation of a novel virus against which there is little pre-existing host immunity [39,40]. As the general population is often relatively immunologically naïve to a shifted viral strain, such a virus may be able to spread rapidly across the globe potentially causing an influenza pandemic [41,42,43,44]. Given that animals, such as birds and pigs, are reservoirs for influenza virus, viral surveillance is an important way to monitor for dangerous viral reassortment [31]. Approaches to monitor influenza virus in animals have been developed, such as the One Health program, in order to better understand how virus can spread to humans thereby affecting public health [45,46]. Containing the spread of influenza virus through vaccination efforts, especially in low- and middle-income countries, is a key way to help protect public health of the population, but in particular children and pregnant women. 

### 2.2. The Host Immune Response to Influenza Virus Infection

**Figure 2:**
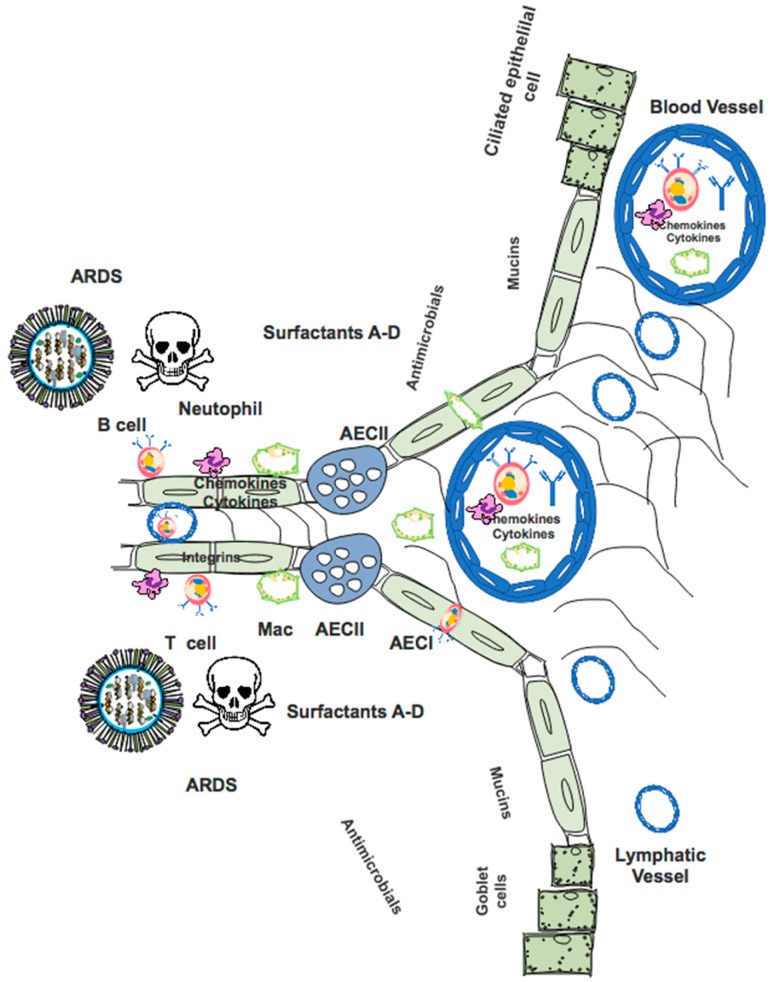
Factors involved in defense against influenza virus infection, which can also contribute to lung damage and ARDS. Cells of the immune system (e.g., macrophages; T cells; B cells; neutrophils) and lung (e.g., alveolar epithelial type I AECI; alveolar epithelial type II AECII; ciliated cells; goblet cells) in addition to factors such as surfactants, mucins, and antimicrobial proteins, interact to protect the host from influenza virus. Direct cytopathic effects of influenza virus infection and excessive inflammation lead to damage of alveoli, which can compromise respiratory function.

The host response to influenza virus can limit viral infection within the lung, thereby protecting host health. However, failure to appropriately regulate this response can lead to damage of lung alveoli due to excessive inflammation or cytolysis of lung cells due to viral infection. The result is impaired gas exchange, which is a major morbidity associated with influenza virus infection. Figure 2 summarizes factors that are responsible for protecting the host against infection with influenza virus. These include epithelial cells, cells of the innate immune system, the adaptive immune system, cytokines, chemokines, antibodies, and surfactant proteins. 

Influenza virus first targets epithelial cells of the proximal respiratory system through HA binding to α2,6-linked sialylated proteins (reviewed in [21,47,48]). Binding and/or internalization of the virus to cells of the epithelium leads to intracellular signaling that alters ion transport, contributing to symptoms of infection [49]. Antiviral responses are initiated, including the release of antimicrobial peptides such as surfactants, mucins, LL-37 and β-defensins, which decrease viral binding to epithelial cells and promote recruitment of innate immune cells such as neutrophils [47,50,51]. Surfactant proteins are capable of binding to virus, which helps to limit infectivity and disease severity [52,53,54,55]. Upon infection, respiratory epithelial cells sense virus through Toll-Like Receptors (TLRs), retinoic acid-inducible gene I (RIG-I), NOD-like receptors (NLRs), and melanoma differentiation-associated 5 (MDA-5), leading to the expression of type-I and type-III interferons (IFN), interleukin-6 (IL-6), IL-1β, IL-18, and other pro-inflammatory cytokines and chemokines [56,57,58,59]. Some of these cytokines and chemokines cause immune cells to extravasate from blood vessels into the site of infection in order to combat the pathogen.

Following influenza virus infection, a classic antiviral response occurs. Among the first cells to become activated are macrophages and dendritic cells, which are critical for the initiation of the antiviral response and instruction of developing adaptive immunity [60,61]. Many subtypes of dendritic cells are present, some of which are capable of presenting antigen to both CD4+ and CD8+ T cells [62]. Delivery of antigen to draining lymph nodes via lymphatic vessels is an important step in activating T cells [63,64]. Dendritic cells are targeted by influenza virus infection, which can impair the development of the adaptive immune response [65]. Once activated, CD8+ T cells will kill virally infected cells, with an important role for lung tissue-resident memory CD8 T cells (T_rm_) in generating rapid antiviral responses upon host reinfection [66,67]. CD4+ T cells also contribute a multiplicity of functions to anti-influenza immunity, including promoting CD8+ T cell function (activation, expansion, positioning, and memory formation), the innate immune response, help for the B cell response, and independent cytotoxicity [68,69]. T follicular helper cells, a specialized CD4+ T cell subset, provide cognate help for both the extrafollicular and germinal center B cell responses [70,71,72]. Antibody secreting cells (ASCs) then home to tissues such as the bone marrow, where they receive survival factors that allow for long term survival, imparting immunity via the high levels of antibody they secrete [73]. An alternate fate upon B cell activation is to become a memory B cell [74,75,76]. In the case of influenza virus, T cells and B cells recognize viral components including both the surface proteins (HA and NA) and the internal virion proteins (NP, NS1, and M1); see Figure 1 [69]. Detectable levels of class-switched antibody are found approximately two weeks following influenza infection [77,78]. These high-affinity antibodies can then act to interfere with viral binding, viral replication, or target infected cells for killing via mechanisms such as antibody-dependent cellular cytotoxicity (ADCC) [79,80]. Following successful viral clearance, cells of the immune system become relatively quiescent once again, leaving a pool of memory T and B cells in addition to protective antibodies that will lead to resistance against future influenza infection [81,82,83]. 

### 2.3. Acute Respiratory Distress Syndrome and Lung Damage as a Consequence of Overwhelming Viral Infection

If the host is unable to control viral infection, respiratory morbidities can occur. One such morbidity is the development of ARDS, which is characterized by pulmonary edema, hypoxemia and a high mortality rate [15]. Infectious agents are just one of many potential causes of ARDS, with both direct influenza virus infection and the anti-influenza immune response contributing to damage of the respiratory tract and ARDS development [17]. In pregnant women and young children, ARDS is a rare but major health concern following infection with influenza virus [14,15], with one study demonstrating influenza virus infection to be a factor contributing to a higher risk of death in pregnant women with ARDS [16]. 

There are many examples of how viral and host factors promote the generation of inflammation in the lung, which is a hallmark of severe influenza-like illness. One report demonstrated that the glycosylation state of HA controlled levels of proinflammatory cytokines produced by human lung epithelial cells [84]. Also, influenza viruses that were selected to infect human epithelial cells became more pathogenic by adaptation to the host, thereby increasing illness severity [85]. It is well known that viral factors, such as NS1, downregulate the production of interferons, thereby decreasing the host antiviral response [86]. While gas-exchanging alveolar type I epithelial cells can generate an antiviral response, high viral loads can overwhelm this and lead to compromised lung function [87,88]. In some severe cases of influenza virus infection, virus and inflammation were noted in the lower respiratory tract or alveoli, with resulting cellular damage (Figure 2) [8,9,11,12,13,89]. Additionally, neutrophils have been implicated in the pathophysiology of alveolar damage following infection with influenza virus [90]. IL-8/CXCL8 and GM-CSF, which are neutrophil chemotactic agents, are produced by primary human alveolar epithelial cells and may exacerbate the inflammatory process in alveoli, thus increasing the likelihood of developing ARDS [91]. TLR3-expressing CD8+ T cells were also found in areas with diffuse alveolar damage in a group of patients who died of severe influenza virus infection [92]. These findings suggest several potential interventions aimed at limiting alveolar damage through control of excessive inflammation that may help to reduce the risk of developing ARDS. 

Controlling viral load and the resulting pulmonary inflammation through vaccination pre-exposure and use of therapeutics post-exposure could help to decrease lung damage and the resulting respiratory morbidity. In support of this, ferrets and mice that were administered human immunoglobulins had a reduction in viral load as compared to control animals that did not [93], with decreased weight loss, decreased mortality, and protection against challenge with H1N1 virus [94]. Similarly, data suggested that a high viral load and a strong cytokine response contributed to mortality in humans infected with H5N1 influenza virus [95]. One recent study demonstrated that children between one and five years of age had higher viral loads compared to older children, perhaps contributing to their increased susceptibility to influenza virus infection [96]. While these results seem to indicate that decreasing viral load would lead to improved outcomes, a recent phase III clinical trial demonstrated that transfer of sera containing high levels of anti-influenza antibody into patients with severe influenza virus infection did not lead to detectable benefit [97]. However, administration of some anti-influenza virus antibodies have been shown to reduce viral loads in humans [98]. Additional work will be required to determine whether antibody treatments will help to reduce influenza virus load and decrease disease severity.

Following clearance of the infection, lung repair occurs via a complicated interplay between the immune system and epithelium [99,100,101,102]. Ineffective repair of alveoli following infection has also been implicated in the development of ARDS [15]. Thus, the interaction between the lung and immune system is the key not only to anti-viral responses, but also for post-infection healing. Unfortunately, basic information regarding the interaction of cells found in human lung is lacking in the field of lung biology. Work from our group is attempting to unravel the crosstalk between epithelial cells and immune cells in the developing human lung [103,104,105]. Such information and the experimental models that have been developed will be useful in future studies aimed at identifying treatments to prevent ARDS or encourage lung healing.

### 2.4. The Pediatric Immune System is Different than that of the Adult

Differences in immunologic function between young children and adults likely contribute to the increased susceptibility of children to influenza virus infection. The immune system of young children is characterized by a higher frequency of naïve antigen-specific cells [106,107]. This population also tends to have an overall higher number of circulating T and B cells that decreases drastically by six years of age [108]. An increase in regulatory T and B cells is also noted in neonatal blood samples [109,110,111,112,113]. Despite the increase in the frequency of regulatory T cells in neonatal blood, the functional ability of neonatal regulatory T cells to suppress dendritic cell function and to blunt the immune reaction is diminished relative to adults [114]. Additionally, cells of the pediatric innate immune system, including monocytes and dendritic cells, tend to be less stimulatory (e.g., increased IL-10 and decreased IL-12, IL-1β, and IFN-α) when challenged with TLR agonists versus adults [115,116]. This leads to decreased antigen presentation and T cell co-stimulation capacity. Neutrophil function, such as the generation of reactive oxygen species and neutrophil extracellular traps, is also reduced in neonates relative to adults [117]. These characteristics of the pediatric immune system may contribute to the increased morbidity of children upon infection with influenza virus and the need for children to receive two doses of IIV vaccine. Efforts are underway to produce adjuvanted vaccines to help prime a more robust immune response (see below).

### 2.5. Alterations in the Immune System and Epithelial Barrier of the Preterm Infants Versus Term Infants

Infants born preterm (<37 weeks gestational age) face a variety of long-term health morbidities. While the threat of developing sepsis is a major concern for all neonates, the concern is even greater for preterm infants [118]. Hyporesponsiveness of monocytes to stimulation and decreased TNF-α responsiveness in non-classical monocyte activation have also been noted in preterm infants, especially prior to 30 weeks gestational age, and may contribute to sepsis risk [119,120]. Infections that are known to cause sepsis compromise the epithelial barrier of the lung and gut, leading to decreased expression of several TLRs and easier access of infectious agents to the underlying tissue, facilitating development of infections [121]. Furthermore, antimicrobial factors such as LL-37 are lower in preterm cord blood versus full term controls, which could be reflective of impaired antimicrobial potential in preterm infants [118]. Work involving our group has shown that preterm B cell responses to the influenza virus vaccine actually generate a higher peak level of influenza virus-specific antibody versus term controls, with levels equalizing by 9 months after vaccination [122,123]. We have also reported that CD4+ T cells found in cord blood from preterm infants who are exposed to inflammatory stimuli *in utero* are more likely to produce proinflammatory cytokines, including IL-6 [124]. These studies are just some examples of studies found in the literature that illustrate how the immune system of preterm infants is different from term infants. 

An additional complication of preterm birth is abnormal lung development. Bronchopulmonary dysplasia (BPD) is a chronic lung disease characterized by inflammation and arrest of alveolar development that affects 30–60% of infants born preterm [125,126,127,128]. Studies suggest that part of the oxidative and mechanical damage is the result of respiratory ventilation [129]. One paper suggests that BPD could predispose infants to developing pediatric ARDS [130]. Of note, a recent study from Australia reports that children with BPD have an increased incidence ratio of being hospitalized due to influenza-related illness (9.0 ratio for children 0–10 years old and 41.6 for the 0–2 year old age range) [131]. Additionally, the length of hospital stay for children with BPD was 7 days longer than for children with cystic fibrosis or other chronic lung diseases. Thus, children who are born preterm and develop BPD are at particularly high risk for developing severe influenza illness. 

### 2.6. Vaccination Strategies for Protecting Public Health Vary Across the World 

Influenza vaccine is administered primarily through intramuscular injection (inactivated subunit or split virus) or spraying intranasally (live attenuated influenza virus). Typically, vaccines contain three or four strains of virus (trivalent or quadrivalent respectively), including two influenza A strains (H1 and H3), and one or two influenza B strains [18]. A vaccine is considered to confer protection if post-vaccination serum can inhibit influenza mediated hemagglutination *in vitro* at a one to forty or greater dilution, although higher HAI titers may be necessary to achieve similar levels of protection in children [132]. Ultimately, the goal of vaccination is to reduce the rate or severity of infection. This can occur by antibodies that interfere with viral binding to target cells (e.g., neutralization) or by inhibiting the “life-cycle” of the virus (e.g., by preventing viral release from infected cells). Antibody-dependent cellular cytotoxicity is another mechanism for killing virally infected cells, which illustrates another important function that antibodies have [79,80]. In addition to antibodies, a recall response of memory CD4+ and CD8+ T cells allows for protection against influenza virus infection [133,134]. 

Recommendations for vaccinating children and pregnant women vary around the world. In the United States and Canada, it is recommended that all infants and children greater than 6 months of age be vaccinated using IIV, with administration of LAIV only in children greater than 24 months of age due to a reported increased risk of post-LAIV wheezing in infants [135,136]. As children may be relatively immunologically naïve to influenza virus, those younger than 9 years of age initially receive a priming dose of vaccine followed by a booster vaccine dose at least 28 days later [136]. In addition, IIV administration is recommended for women who are or will be pregnant during the influenza season [137]. Although the immune system of preterm infants is relatively immature, immunizations are typically given at the same chronological age as in term infants [138,139]. 

Despite broad recommendations for influenza vaccination in some countries, the World Health Organization (WHO) reported that in 2014, only 59% of member countries had vaccination programs in place [140]. Given that 41% of countries did not have vaccination programs in place, the chances for the spread of influenza virus is quite high. Viral reassortment can occur in birds and pigs, which has contributed to the generation of novel pandemic influenza virus strains [141,142]. By decreasing the number of people infected via routine vaccination, there is likely to be a decreased chance for coinfection of birds and pigs with human and animal influenza virus, thus decreasing the chances of reassortment mutants that could become pandemic. Furthermore, if a country has a vaccination plan in place, rapid administration of vaccines in the case of a pandemic would be possible [143]. Amongst members of the European Union there is great variability in influenza vaccination requirements [144], with a set goal of a 75% vaccination rate in the high-risk population, but actual vaccination rates that are much lower [145]. Countries with low- to middle-income typically have low vaccination rates against influenza virus [146]. Individuals living in these countries face a higher disease burden than individuals from higher-income countries [147]. In countries that do have influenza immunization programs, fewer than half recommended vaccinating pregnant women and less than a third recommended routine vaccination of children [140]. Regardless of recommendations, influenza vaccination rates remain suboptimal and demonstrate substantial variability by age, location, and season. In the US, the goal is to vaccinate 70% of the population, but only around 40% of adults were vaccinated each year between 2010 and 2016 [148]. Similarly, one Canadian study reported that, on average, only 29% of respondents had been vaccinated in a given year between the 2006 and 2013 influenza seasons [149]. Furthermore, one study shows that less than half of pregnant women in the United States were vaccinated over three consecutive influenza seasons [150]. Highlighting the importance of vaccination, a study of 358 laboratory-confirmed influenza-associated pediatric deaths between 2010 and 2014 reported that vaccination coverage was low (26% overall and only 31% of high-risk patients) [151]. Unfortunately, children delivered prematurely have higher health risks associated with birth during the influenza season and a higher risk for being admitted into the hospital than full-term children [131,152,153], yet a recent study demonstrated that late preterm infants were less likely than term infants to be appropriately vaccinated against influenza by 36 months of age [154]. These data highlight the importance of efforts to increase overall influenza vaccination rates among both pregnant women and children. 

### 2.7. A Key Role for Maternal Vaccination in Protecting Young Infants Against Influenza 

The World Health Organization has identified pregnant women as a priority group for receiving influenza vaccines [155]. Pregnant women are particularly susceptible to developing severe influenza illness and have an increased risk for hospital admission, although the mortality rate compared to non-pregnant women may not be increased [156,157,158]. Overall, it has been established that vaccinating pregnant women is safe, with no evidence demonstrating a link between immunization during pregnancy and adverse outcomes in offspring [159,160,161,162,163,164,165]. Infants born to mothers who were vaccinated against influenza may have a decreased rate of preterm birth, low birthweight, and stillborn birth, although establishing a causal relationship is challenging [160,166,167,168,169,170,171]. Infants born to mothers who were severely sick with H1N1 influenza infection have been shown to have an increased risk of preterm birth, decreased 5 min Apgar scores, and an increased risk of death [172,173,174]. These data support vaccinating pregnant women to help improve the health of both the mother and child.

Benefits to the fetus from maternal vaccination against influenza virus include transfer of maternal antibody through the placenta. This is critical for protecting neonates and infants less than six months of age against severe influenza virus infection requiring hospitalization, as these infants are too young to be vaccinated [7,153]. Studies have demonstrated that some isotypes of anti-influenza antibodies cross the placenta and result in higher HAI titers in cord blood compared to placebo recipients [175], with protective antibody transported to the fetus in as little as two weeks post-vaccination [176]. Transplacental transport of antibodies requires expression of the neonatal Fc receptor expression, with IgG, especially IgG1, being effectively transported into fetal circulation [177,178]. Importantly, decreased rates of acute lower respiratory tract infection, influenza virus infections, and influenza-related hospitalizations were reported in infants following maternal vaccination during pregnancy [179,180,181]. While vaccination at any point during pregnancy is recommended, children born to mothers who were vaccinated more than 4 weeks prior to delivery and during the second or third trimester had higher antibody titers against the A(H1N1) virus when compared to those who were born to mother that were vaccinated in the first trimester [182]. 

Another benefit of maternal vaccination is transfer of protective antibodies to children via breastmilk. This is particularly important for antibodies of the IgA isotype, which are not passed through the placenta [183,184]. Vaccination of breastfeeding mothers with IIV resulted in significantly higher HAI titers in serum and IgG and IgA levels in breast milk as compared to vaccination using LAIV, suggesting that IIV may be the preferred vaccine for use in breastfeeding mothers [185]. Given that preterm infants have lower levels of IgG antibody transferred via the placenta, antibody transferred via breastmilk may be of particular importance to decrease influenza virus infection in the preterm population [186,187]. Of note, milk from mothers who delivered prematurely had lower levels of total IgG and IgM but not IgA as compared to mothers who delivered at term [186]. Increasingly, preterm infants are being fed human donor breast milk, which contains significantly lower concentrations of IgM than non-donor milk, possibly due to the pasteurization process. However, administration of any antibody through donor breast milk would logically be better than receiving none at all. Several recent studies have focused on antibody transfer to the stomachs of preterm infants. Antibodies have been shown to be more stable in the gastric contents of preterm infants versus term infants [186], with influenza-virus-specific IgA antibodies being more stable in gastric contents of preterm infants regardless of whether milk is from the birth mother or from a donor [188]. 

In addition to antibodies, other bioactive molecules are found in breast milk, including cytokines and human milk oligosaccharides (HMOs) that have been shown to inhibit viral entry into cells [189,190,191]. Furthermore, cells are transferred to the infant via breastfeeding, which could play a role in shaping the neonatal and infant microbiome [192]. Of note, studies have shown that HMOs fed to mice could be transferred into circulation, had no observable adverse health effects and improved the immune response to influenza virus infection [193,194,195]. In adult humans, HMO ingestion was well tolerated in a two-week oral administration regiment [196]. Thus, breast milk offers a variety of factors in addition to antibodies that help to protect offspring against influenza virus infection. Taken together, the above factors contribute to improved maternal and fetal health afforded by vaccinating pregnant women against influenza virus.

### 2.8. Adjuvanted Influenza Vaccines

Due to the overall poor immunogenicity of influenza vaccines, the use of immune adjuvants is being evaluated as a strategy to increase vaccine immunogenicity. Several different adjuvants have been tested in pre-clinical models and clinical trials. These include aluminum salt (alum) based approaches, oil and water emulsions (MF59, AS03, and AF03), innate immune cell receptor agonists (e.g., TLRs and the inflammasome), and virosomes (lipid bilayer droplets) [197]. These adjuvants utilize multiple mechanisms to increase the immunogencity of influenza vaccines, including activating antigen-presenting cells (APC), increasing antigen uptake by APCs, and recruiting immune cells to the site of vaccine administration [115,116]. Several clinical trials have been conducted in adults and children indicate that the use of AS03 as an adjuvant in influenza vaccines and increases influenza-virus-specific antibody response [198,199,200,201,202,203,204]. Clinical trials using MF-59 as an adjuvant also demonstrated safety and improved rates of seroconversion in preterm and term infants [205], with more robust antiviral responses, activation of dendritic cells and an increased CD4+ T cell cytokine response following administration of an adjuvanted vaccine [206,207,208]. MF59 has been approved for use in the United States since 2015 and its use appears to not increase adverse reaction rates [209]. Additionally, several studies have examined the use of adjuvanted H1N1 vaccines in pregnant women and have found that these vaccines were well tolerated [210,211,212]. Although the increased immunogenicity is strongly advantageous, there is concern for potential adverse effects, including a possible association between an AS03-adjuvanted monovalent pandemic influenza vaccine and narcolepsy [213,214,215]. A recent paper reports a higher rate of adverse events reported when the vaccine was administered to patients outside of the recommended age groups [209]. Reports of adverse events have likely slowed the uptake of adjuvanted influenza vaccines in vulnerable populations such as pregnant women and children. 

### 2.9. Increasing Vaccination Rates for Influenza

Vaccines must be considered effective at preventing disease and be regarded as safe [216]. However, adverse reactions do occur and can include fever, febrile seizures, hypersensitivity reactions, and possibly a small increase in the risk of developing Guillain-Barre syndrome [217,218]. Several organizations, including the WHO, the European Union, the US government, and independent agencies have mechanisms in place for tracking vaccine safety [216,219,220]. In the United States, the Vaccine Adverse Event Reporting System passively documents post-licensure adverse events following vaccination administration [221]. In Europe, the European Medicines Agency is responsible for tracking adverse events through the EudraVigilance Program [222]. Such data are valuable, however, a deeper understanding of additional factors that could have contributed to an adverse event is necessary in order to minimize the risk of drawing an invalid conclusion associating an adverse event with vaccination. Vaccinating pregnant women is generally regarded as safe [159,160,161,162,163,164,165]. However, some hesitancy over influenza vaccination is present in this population and the benefit of protecting both the mother and the fetus needs to be conveyed as outweighing the risk of potential side effects [223,224,225,226].

Perhaps not surprisingly, preemptive action is more cost-effective than is reactive action for a pandemic, although modeling suggests mortality rates don’t differ between preemptive and reactive responses [227]. It is estimated that in order to prevent one case of influenza, five individuals would need to be vaccinated with IIV and seven with LAIV [9]. The importance of having a well-vaccinated population is at the heart of “herd immunity”, where disruption of influenza transmission will lead to a less severe outbreak [228]. Herd immunity in children has been modeled, with studies suggesting that a substantial decrease in influenza infection of non-vaccinated individuals is predicted to occur, but only at very high rates of vaccine coverage (i.e., 90%) [229]. Development of more effective vaccines and increasing the rate of vaccination will bring the population closer protection from influenza virus infection [230]. Given the strong benefit of vaccinating pregnant women and children and the low vaccination rates across the globe, finding effective ways to increase these vaccination rates is critical for improving public health.

One major reason for vaccine refusal relates to the public’s lack of trust in the pharmaceutical industry, medical providers, and efficacy of the influenza vaccine [231]. Some work suggests that mandatory vaccination increases negative feelings and anger regarding vaccines as compared to voluntary vaccination programs [232]. Such feelings could also lead to a stronger aversion to all vaccines [233]. A particularly influential factor that has led to mistrust of vaccine safety has been rooted in the belief that vaccination is associated with children being placed on the autism spectrum [234]. Initial studies making this claim have since been retracted, and subsequent work has not supported this association [235,236]. Despite this fact, recent studies continue to demonstrate that younger siblings of children on the autism spectrum disorder are not fully vaccinated [237,238]. This work illustrates the long-lasting harm caused by the initial reports linking vaccine administration to autism. Furthermore, it shows that the general public is still very much concerned with the perceived risk of administering vaccines to children despite a lack of supporting evidence. Rebuilding confidence in vaccines will require communication and trust between clinical providers and patients [239,240].

Increasing vaccination rates in low- and middle-income countries will require additional resources to help offset the cost of development and implementation of vaccination programs [225]. It has been estimated that global supply of influenza virus vaccine is sufficient to cover pregnant women in low- and middle-income countries [241]. A recent phase 4 clinical trial demonstrated that developing a vaccination program for pregnant women is possible in Mali, a low-income country [242,243]. While the Mali study was not sufficiently powered to detect a decrease in ILI, maternal vaccination studies conducted in Bangladesh and South Africa have demonstrated at least partial protection against developing confirmed influenza virus infection [175,180]. Unfortunately, due to the lack of sufficient data for the benefit of developing such programs, the Global Alliance for Vaccines and Immunization has deprioritized maternal vaccinations against seasonal flu [244,245,246,247] while the Global Influenza Initiative recommends that all pregnant women be vaccinated in their third trimester [225]. This lack of harmonization between guidelines highlights the additional work needed to develop evidence-based recommendations for programs in resource poor countries [248]. Regardless, by increasing vaccine uptake in low- and middle-income countries, not only will human health be improved, but the chances of virus spreading to an expanded geographical region are lessened. This will help to contain virus and likely decrease the chance of establishing a pandemic.

## 3. Discussion

Influenza infection is a serious health concern, especially for pregnant women and young children. While influenza vaccines are generally considered to be safe, vaccine uptake remains suboptimal. Vaccination of pregnant women provides protection against influenza infection in both the expectant mother as well as the infant due to transplacental transfer of influenza-virus-specific IgG antibody. Additionally, breastfeeding provides antibodies (in particular IgA that is not passed through the placenta) and immunomodulatory factors to prevent and/or combat influenza infection. These factors are particularly important in the first six months of life, as active vaccination is not recommended for this age group. For infants born prematurely, complicating factors such as chronic lung disease increases the risk for developing severe illness after influenza infection. Despite having an immature immune system, vaccination is recommended on schedule in this population and has been shown to be protective. Furthermore, vaccination of these populations will help to guard against the development of ARDS, which is a major health concern following infection with influenza virus.

Influenza viruses undergoes continuous antigenic drift, which leads to lower than ideal vaccine efficacy in some seasons. Furthermore, antigenic shifts resulting in pandemic outbreaks is not uncommon, with 4 pandemics in the 21st century. Next generation influenza vaccines targeted against highly conserved regions of the influenza virus are being developed that may provide more universal protection against even potentially pandemic influenza strains. However, even if conventional vaccine strains of influenza are not well matched to circulating strains, reduced viral shedding and a shorter duration and severity of illness are often observed in the vaccinated population. Such information must be effectively disseminated to members of the public in order to improve vaccine uptake, as misconceptions about influenza vaccine adverse effects and effectiveness remains major obstacles to improving worldwide influenza vaccination rates. 

## 4. Conclusions

Influenza vaccination reduces the risk of influenza infection, severe disease, morbidity, and death. Despite this, the rate of influenza vaccination remains well below targets set forth by international and national health officials. While improving vaccination rates, especially among pregnant women and young children, is a high priority, additional large-scale studies would be of benefit to generate evidence-based recommendations in support of existing programs and to increase public confidence in current recommendations. In addition, development of more immunogenic and universally protective influenza vaccines will increase the breadth of protection provided while decreasing the frequency of vaccination required. Such efforts will be critical to increasing uptake of influenza vaccines and will improve the health of at-risk populations, such as children and pregnant women.

## Abbreviations

ARDS	Acute respiratory distress syndrome
IIV	Inactivated Influenza Vaccine
LAIV	Live Attenuated Influenza Virus
HA	hemagglutinin
NA	neuraminidase
NP	nucleoprotein
M1	matrix protein 1
M2/BM2	matrix protein 2
NS1	nonstructural protein 1
NS2	nonstructural protein 2
TLRs	Toll-Like Receptors
RIG-I	retinoic acid-inducible gene I
NLRs	NOD-like receptors
MDA-5	melanoma differentiation-associated 5
IFN	interferons
IL	interleukin
ASCs	antibody secreting cells
ADCC	antibody-dependent cellular cytotoxicity
WHO	World Health Organization
AECI	alveolar epithelial type I
AECII	alveolar epithelial type II

## References

[1] Doyon-Plourde P., Fakih I., Tadount F., Fortin E., Quach C. (2019). Impact of influenza vaccination on healthcare utilization—A systematic review. Vaccine.

[2] United_States_Food_and_Drug_Administration Pediatric Medical Devices. https://www.fda.gov/medical-devices/products-and-medical-procedures/pediatric-medical-devices.

[3] Nair H., Brooks W.A., Katz M., Roca A., Berkley J.A., Madhi S.A., Simmerman J.M., Gordon A., Sato M., Howie S. (2011). Global burden of respiratory infections due to seasonal influenza in young children: A systematic review and meta-analysis. Lancet.

[4] Nunes M.C., Madhi S.A. (2018). Influenza vaccination during pregnancy for prevention of influenza confirmed illness in the infants: A systematic review and meta-analysis. Hum. Vaccines Immunother..

[5] Lafond K.E., Nair H., Rasooly M.H., Valente F., Booy R., Rahman M., Kitsutani P., Yu H., Guzman G., Coulibaly D. (2016). Global Role and Burden of Influenza in Pediatric Respiratory Hospitalizations, 1982–2012: A Systematic Analysis. PLoS Med..

[6] Committee On Infectious Diseases (2018). Recommendations for Prevention and Control of Influenza in Children, 2018–2019. Pediatrics.

[7] Nunes M.C., Madhi S.A. (2018). Prevention of influenza-related illness in young infants by maternal vaccination during pregnancy. F1000Research.

[8] Short K.R., Kroeze E., Fouchier R.A.M., Kuiken T. (2014). Pathogenesis of influenza-induced acute respiratory distress syndrome. Lancet Infect. Dis..

[9] Simonsen L., Clarke M.J., Schonberger L.B., Arden N.H., Cox N.J., Fukuda K. (1998). Pandemic versus epidemic influenza mortality: A pattern of changing age distribution. J. Infect. Dis..

[10] Short K.R., Kasper J., van der Aa S., Andeweg A.C., Zaaraoui-Boutahar F., Goeijenbier M., Richard M., Herold S., Becker C., Scott D.P. (2016). Influenza virus damages the alveolar barrier by disrupting epithelial cell tight junctions. Eur. Respir. J..

[11] Wonderlich E.R., Swan Z.D., Bissel S.J., Hartman A.L., Carney J.P., O’Malley K.J., Obadan A.O., Santos J., Walker R., Sturgeon T.J. (2017). Widespread Virus Replication in Alveoli Drives Acute Respiratory Distress Syndrome in Aerosolized H5N1 Influenza Infection of Macaques. J. Immunol..

[12] Kennedy E.D., Roy M., Norris J., Fry A.M., Kanzaria M., Blau D.M., Shieh W.J., Zaki S.R., Waller K., Kamimoto L. (2013). Lower respiratory tract hemorrhage associated with 2009 pandemic influenza A (H1N1) virus infection. Influenza Other Respir. Viruses.

[13] Shieh W.J., Blau D.M., Denison A.M., Deleon-Carnes M., Adem P., Bhatnagar J., Sumner J., Liu L., Patel M., Batten B. (2010). 2009 pandemic influenza A (H1N1): Pathology and pathogenesis of 100 fatal cases in the United States. Am. J. Pathol..

[14] Nye S., Whitley R.J., Kong M. (2016). Viral Infection in the Development and Progression of Pediatric Acute Respiratory Distress Syndrome. Front. Pediatr..

[15] Matthay M.A., Zemans R.L., Zimmerman G.A., Arabi Y.M., Beitler J.R., Mercat A., Herridge M., Randolph A.G., Calfee C.S. (2019). Acute respiratory distress syndrome. Nat. Rev. Dis Primers.

[16] Rush B., Martinka P., Kilb B., McDermid R.C., Boyd J.H., Celi L.A. (2017). Acute Respiratory Distress Syndrome in Pregnant Women. Obstet. Gynecol..

[17] Kalil A.C., Thomas P.G. (2019). Influenza virus-related critical illness: Pathophysiology and epidemiology. Crit. Care.

[18] Mameli C., Cocchi I., Fumagalli M., Zuccotti G. (2019). Influenza Vaccination: Effectiveness, Indications, and Limits in the Pediatric Population. Front. Pediatri..

[19] De A. (2018). Molecular evolution of hemagglutinin gene of Influenza A virus. Front. Biosci..

[20] Arai Y., Kawashita N., Hotta K., Hoang P.V.M., Nguyen H.L.K., Nguyen T.C., Vuong C.D., Le T.T., Le M.T.Q., Soda K. (2018). Multiple polymerase gene mutations for human adaptation occurring in Asian H5N1 influenza virus clinical isolates. Sci. Rep..

[21] de Graaf M., Fouchier R.A. (2014). Role of receptor binding specificity in influenza A virus transmission and pathogenesis. EMBO J..

[22] Goka E.A., Vallely P.J., Mutton K.J., Klapper P.E. (2014). Mutations associated with severity of the pandemic influenza A(H1N1)pdm09 in humans: A systematic review and meta-analysis of epidemiological evidence. Arch. Virol..

[23] Simon B., Pichon M., Valette M., Burfin G., Richard M., Lina B., Josset L. (2019). Whole Genome Sequencing of A(H3N2) Influenza Viruses Reveals Variants Associated with Severity during the 2016–2017 Season. Viruses.

[24] Arai Y., Kawashita N., Ibrahim M.S., Elgendy E.M., Daidoji T., Ono T., Takagi T., Nakaya T., Matsumoto K., Watanabe Y. (2019). PB2 mutations arising during H9N2 influenza evolution in the Middle East confer enhanced replication and growth in mammals. PLoS Pathog..

[25] Seladi-Schulman J., Campbell P.J., Suppiah S., Steel J., Lowen A.C. (2014). Filament-producing mutants of influenza A/Puerto Rico/8/1934 (H1N1) virus have higher neuraminidase activities than the spherical wild-type. PLoS ONE.

[26] Wang Q., Bhattacharya S., Mereness J.A., Anderson C., Lillis J.A., Misra R.S., Romas S., Huyck H., Howell A., Bandyopadhyay G. (2019). A novel in vitro model of primary human pediatric lung epithelial cells. Pediatr. Res..

[27] Thangavel R.R., Reed A., Norcross E.W., Dixon S.N., Marquart M.E., Stray S.J. (2011). “Boom” and “Bust” cycles in virus growth suggest multiple selective forces in influenza a evolution. Virol. J..

[28] Zhou F., Trieu M.C., Davies R., Cox R.J. (2018). Improving influenza vaccines: Challenges to effective implementation. Curr. Opin. Immunol..

[29] Valkenburg S.A., Leung N.H.L., Bull M.B., Yan L.M., Li A.P.Y., Poon L.L.M., Cowling B.J. (2018). The Hurdles From Bench to Bedside in the Realization and Implementation of a Universal Influenza Vaccine. Front. Immunol..

[30] Epstein S.L. (2018). Universal Influenza Vaccines: Progress in Achieving Broad Cross-Protection In Vivo. Am. J. Epidemiol..

[31] Krammer F., Smith G.J.D., Fouchier R.A.M., Peiris M., Kedzierska K., Doherty P.C., Palese P., Shaw M.L., Treanor J., Webster R.G. (2018). Influenza. Nat. Rev. Dis. Primers.

[32] Sharma L., Rebaza A., Dela Cruz C.S. (2019). When “B” becomes “A”: The emerging threat of influenza B virus. Eur. Respir. J..

[33] Tan J., Asthagiri Arunkumar G., Krammer F. (2018). Universal influenza virus vaccines and therapeutics: Where do we stand with influenza B virus?. Curr. Opin. Immunol..

[34] Proff R., Gershman K., Lezotte D., Nyquist A.C. (2009). Case-based surveillance of influenza hospitalizations during 2004–2008, Colorado, USA. Emerg. Infect. Dis..

[35] Chiu S.S., Chan K.H., Chen H., Young B.W., Lim W., Wong W.H., Lau Y.L., Peiris J.S. (2009). Virologically confirmed population-based burden of hospitalization caused by influenza A and B among children in Hong Kong. Clin. Infect. Dis..

[36] Tran D., Vaudry W., Moore D., Bettinger J.A., Halperin S.A., Scheifele D.W., Jadvji T., Lee L., Mersereau T., Members of the Canadian Immunization Monitoring Program Active (2016). Hospitalization for Influenza A Versus B. Pediatrics.

[37] Schrauwen E.J., de Graaf M., Herfst S., Rimmelzwaan G.F., Osterhaus A.D., Fouchier R.A. (2014). Determinants of virulence of influenza A virus. Eur. J. Clin. Microbiol. Infect. Dis..

[38] Wang H., Dou D., Ostbye H., Revol R., Daniels R. (2019). Structural restrictions for influenza neuraminidase activity promote adaptation and diversification. Nat. Microbiol..

[39] Morens D.M., Taubenberger J.K., Fauci A.S. (2009). The persistent legacy of the 1918 influenza virus. N. Engl. J. Med..

[40] Lowen A.C. (2017). Constraints, Drivers, and Implications of Influenza A Virus Reassortment. Annu. Rev. Virol..

[41] Potter C.W., Jennings R. (2011). A definition for influenza pandemics based on historical records. J. Infect..

[42] Werner J., Schudrowitz C., Kohler H. (1975). Antigenic variation of neuraminidase of human type A influenza (H3N2) viruses isolated in Berlin (West). Zentralbl Bakteriol Orig A.

[43] Kelly H. (2011). The classical definition of a pandemic is not elusive. Bull. World Health Organ..

[44] Doshi P. (2011). The elusive definition of pandemic influenza. Bull. World Health Organ..

[45] Zinsstag J., Schelling E., Waltner-Toews D., Tanner M. (2011). From “one medicine” to “one health” and systemic approaches to health and well-being. Prev. Vet. Med..

[46] Short K.R., Richard M., Verhagen J.H., van Riel D., Schrauwen E.J., van den Brand J.M., Manz B., Bodewes R., Herfst S. (2015). One health, multiple challenges: The inter-species transmission of influenza A virus. One Health.

[47] Denney L., Ho L.P. (2018). The role of respiratory epithelium in host defence against influenza virus infection. Biomed. J..

[48] Luo M. (2012). Influenza virus entry. Adv. Exp. Med. Biol..

[49] Londino J.D., Lazrak A., Collawn J.F., Bebok Z., Harrod K.S., Matalon S. (2017). Influenza virus infection alters ion channel function of airway and alveolar cells: Mechanisms and physiological sequelae. Am. J. Physiol. Lung Cell. Mol. Physiol..

[50] Chen Z.G., Wang Z.N., Yan Y., Liu J., He T.T., Thong K.T., Ong Y.K., Chow V.T.K., Tan K.S., Wang Y. (2019). Upregulation of cell-surface mucin MUC15 in human nasal epithelial cells upon influenza A virus infection. BMC Infect. Dis..

[51] Hsieh I.N., De Luna X., White M.R., Hartshorn K.L. (2018). The Role and Molecular Mechanism of Action of Surfactant Protein D in Innate Host Defense Against Influenza A Virus. Front. Immunol..

[52] Nikolaidis N.M., White M.R., Allen K., Tripathi S., Qi L., McDonald B., Taubenberger J., Seaton B.A., McCormack F.X., Crouch E.C. (2014). Mutations flanking the carbohydrate binding site of surfactant protein D confer antiviral activity for pandemic influenza A viruses. Am. J. Physiol. Lung Cell. Mol. Physiol..

[53] Herrera-Ramos E., Lopez-Rodriguez M., Ruiz-Hernandez J.J., Horcajada J.P., Borderias L., Lerma E., Blanquer J., Perez-Gonzalez M.C., Garcia-Laorden M.I., Florido Y. (2014). Surfactant protein A genetic variants associate with severe respiratory insufficiency in pandemic influenza A virus infection. Crit. Care.

[54] Benne C.A., Kraaijeveld C.A., van Strijp J.A., Brouwer E., Harmsen M., Verhoef J., van Golde L.M., van Iwaarden J.F. (1995). Interactions of surfactant protein A with influenza A viruses: Binding and neutralization. J. Infect. Dis..

[55] To K.K.W., Zhou J., Song Y.Q., Hung I.F.N., Ip W.C.T., Cheng Z.S., Chan A.S.F., Kao R.Y.T., Wu A.K.L., Chau S. (2014). Surfactant protein B gene polymorphism is associated with severe influenza. Chest.

[56] Hamilton J.R., Sachs D., Lim J.K., Langlois R.A., Palese P., Heaton N.S. (2016). Club cells surviving influenza A virus infection induce temporary nonspecific antiviral immunity. Proc. Natl. Acad. Sci. USA.

[57] Chamberlain N., Korwin-Mihavics B.R., Nakada E.M., Bruno S.R., Heppner D.E., Chapman D.G., Hoffman S.M., van der Vliet A., Suratt B.T., Dienz O. (2019). Lung epithelial protein disulfide isomerase A3 (PDIA3) plays an important role in influenza infection, inflammation, and airway mechanics. Redox Biol..

[58] Sell S., McKinstry K.K., Strutt T.M. (2019). Mouse Models Reveal Role of T-Cytotoxic and T-Reg Cells in Immune Response to Influenza: Implications for Vaccine Design. Viruses.

[59] Kim T.H., Lee H.K. (2014). Differential roles of lung dendritic cell subsets against respiratory virus infection. Immune Netw..

[60] He W., Chen C.J., Mullarkey C.E., Hamilton J.R., Wong C.K., Leon P.E., Uccellini M.B., Chromikova V., Henry C., Hoffman K.W. (2017). Alveolar macrophages are critical for broadly-reactive antibody-mediated protection against influenza A virus in mice. Nat. Commun..

[61] Kumagai Y., Takeuchi O., Kato H., Kumar H., Matsui K., Morii E., Aozasa K., Kawai T., Akira S. (2007). Alveolar macrophages are the primary interferon-alpha producer in pulmonary infection with RNA viruses. Immunity.

[62] Vangeti S., Yu M., Smed-Sorensen A. (2018). Respiratory Mononuclear Phagocytes in Human Influenza A Virus Infection: Their Role in Immune Protection and As Targets of the Virus. Front. Immunol..

[63] Spitaels J., Roose K., Saelens X. (2016). Influenza and Memory T Cells: How to Awake the Force. Vaccines.

[64] Lambrecht B.N., Hammad H. (2012). Lung dendritic cells in respiratory viral infection and asthma: From protection to immunopathology. Annu. Rev. Immunol..

[65] Smed-Sorensen A., Chalouni C., Chatterjee B., Cohn L., Blattmann P., Nakamura N., Delamarre L., Mellman I. (2012). Influenza A virus infection of human primary dendritic cells impairs their ability to cross-present antigen to CD8 T cells. PLoS Pathog..

[66] Pizzolla A., Nguyen T.H.O., Smith J.M., Brooks A.G., Kedzierska K., Heath W.R., Reading P.C., Wakim L.M. (2017). Resident memory CD8+ T cells in the upper respiratory tract prevent pulmonary influenza virus infection. Sci. Immunol..

[67] Wu T., Hu Y., Lee Y.T., Bouchard K.R., Benechet A., Khanna K., Cauley L.S. (2014). Lung-resident memory CD8 T cells (TRM) are indispensable for optimal cross-protection against pulmonary virus infection. J. Leukoc. Biol..

[68] Sant A.J., Richards K.A., Nayak J. (2018). Distinct and complementary roles of CD4 T cells in protective immunity to influenza virus. Curr. Opin. Immunol..

[69] Sant A.J., DiPiazza A.T., Nayak J.L., Rattan A., Richards K.A. (2018). CD4 T cells in protection from influenza virus: Viral antigen specificity and functional potential. Immunol. Rev..

[70] Biram A., Davidzohn N., Shulman Z. (2019). T cell interactions with B cells during germinal center formation, a three-step model. Immunol. Rev..

[71] Takahashi Y., Onodera T., Adachi Y., Ato M. (2017). Adaptive B Cell Responses to Influenza Virus Infection in the Lung. Viral Immunol..

[72] Shinnakasu R., Kurosaki T. (2017). Regulation of memory B and plasma cell differentiation. Curr. Opin. Immunol..

[73] Nguyen D.C., Garimalla S., Xiao H., Kyu S., Albizua I., Galipeau J., Chiang K.Y., Waller E.K., Wu R., Gibson G. (2018). Factors of the bone marrow microniche that support human plasma cell survival and immunoglobulin secretion. Nat. Commun..

[74] Lavine S.D., Cockroft K., Hoh B., Bambakidis N., Khalessi A.A., Woo H., Riina H., Siddiqui A., Hirsch J.A., Chong W. (2017). Erratum to: Training guidelines for endovascular stroke intervention: An international multi-society consensus document. Neuroradiology.

[75] Ellebedy A.H., Jackson K.J., Kissick H.T., Nakaya H.I., Davis C.W., Roskin K.M., McElroy A.K., Oshansky C.M., Elbein R., Thomas S. (2016). Defining antigen-specific plasmablast and memory B cell subsets in human blood after viral infection or vaccination. Nat. Immunol..

[76] Good-Jacobson K.L. (2018). Strength in diversity: Phenotypic, functional, and molecular heterogeneity within the memory B cell repertoire. Immunol. Rev..

[77] Ellebedy A.H. (2018). Immunizing the Immune: Can We Overcome Influenza’s Most Formidable Challenge?. Vaccines.

[78] Lam J.H., Baumgarth N. (2019). The Multifaceted B Cell Response to Influenza Virus. J. Immunol..

[79] Padilla-Quirarte H.O., Lopez-Guerrero D.V., Gutierrez-Xicotencatl L., Esquivel-Guadarrama F. (2019). Protective Antibodies Against Influenza Proteins. Front. Immunol..

[80] Vanderven H.A., Jegaskanda S., Wheatley A.K., Kent S.J. (2017). Antibody-dependent cellular cytotoxicity and influenza virus. Curr. Opin. Virol..

[81] DiPiazza A., Richards K.A., Knowlden Z.A., Nayak J.L., Sant A.J. (2016). The Role of CD4 T Cell Memory in Generating Protective Immunity to Novel and Potentially Pandemic Strains of Influenza. Front. Immunol..

[82] Dhume K., McKinstry K.K. (2018). Early programming and late-acting checkpoints governing the development of CD4 T-cell memory. Immunology.

[83] Auladell M., Jia X., Hensen L., Chua B., Fox A., Nguyen T.H.O., Doherty P.C., Kedzierska K. (2019). Recalling the Future: Immunological Memory Toward Unpredictable Influenza Viruses. Front. Immunol..

[84] Hrincius E.R., Liedmann S., Finkelstein D., Vogel P., Gansebom S., Samarasinghe A.E., You D., Cormier S.A., McCullers J.A. (2015). Acute Lung Injury Results from Innate Sensing of Viruses by an ER Stress Pathway. Cell Rep..

[85] Huang D.T., Lu C.Y., Chi Y.H., Li W.L., Chang L.Y., Lai M.J., Chen J.S., Hsu W.M., Huang L.M. (2017). Adaptation of influenza A (H7N9) virus in primary human airway epithelial cells. Sci. Rep..

[86] Krug R.M. (2015). Functions of the influenza A virus NS1 protein in antiviral defense. Curr. Opin. Virol..

[87] Sanders C.J., Vogel P., McClaren J.L., Bajracharya R., Doherty P.C., Thomas P.G. (2013). Compromised respiratory function in lethal influenza infection is characterized by the depletion of type I alveolar epithelial cells beyond threshold levels. Am. J. Physiol. Lung Cell. Mol. Physiol..

[88] Rosenberger C.M., Podyminogin R.L., Askovich P.S., Navarro G., Kaiser S.M., Sanders C.J., McClaren J.L., Tam V.C., Dash P., Noonan J.G. (2014). Characterization of innate responses to influenza virus infection in a novel lung type I epithelial cell model. J. Gen. Virol..

[89] Gisslen T., Alvarez M., Wells C., Soo M.T., Lambers D.S., Knox C.L., Meinzen-Derr J.K., Chougnet C.A., Jobe A.H., Kallapur S.G. (2016). Fetal inflammation associated with minimal acute morbidity in moderate/late preterm infants. Archives of disease in childhood. Fetal Neonatal Ed..

[90] Tang B.M., Shojaei M., Teoh S., Meyers A., Ho J., Ball T.B., Keynan Y., Pisipati A., Kumar A., Eisen D.P. (2019). Neutrophils-related host factors associated with severe disease and fatality in patients with influenza infection. Nat. Commun..

[91] Ito Y., Correll K., Zemans R.L., Leslie C.C., Murphy R.C., Mason R.J. (2015). Influenza induces IL-8 and GM-CSF secretion by human alveolar epithelial cells through HGF/c-Met and TGF-alpha/EGFR signaling. Am. J. Physiol. Lung Cell. Mol. Physiol..

[92] Mauad T., Hajjar L.A., Callegari G.D., da Silva L.F., Schout D., Galas F.R., Alves V.A., Malheiros D.M., Auler J.O., Ferreira A.F. (2010). Lung pathology in fatal novel human influenza A (H1N1) infection. Am. J. Respir. Crit. Care Med..

[93] Rockman S., Lowther S., Camuglia S., Vandenberg K., Taylor S., Fabri L., Miescher S., Pearse M., Middleton D., Kent S.J. (2017). Intravenous Immunoglobulin Protects Against Severe Pandemic Influenza Infection. EBioMedicine.

[94] Hohenadl C., Wodal W., Kerschbaum A., Fritz R., Howard M.K., Farcet M.R., Portsmouth D., McVey J.K., Baker D.A., Ehrlich H.J. (2014). Hyperimmune intravenous immunoglobulin containing high titers of pandemic H1N1 hemagglutinin and neuraminidase antibodies provides dose-dependent protection against lethal virus challenge in SCID mice. Virol. J..

[95] de Jong M.D., Simmons C.P., Thanh T.T., Hien V.M., Smith G.J., Chau T.N., Hoang D.M., Chau N.V., Khanh T.H., Dong V.C. (2006). Fatal outcome of human influenza A (H5N1) is associated with high viral load and hypercytokinemia. Nat. Med..

[96] Roosenhoff R., Reed V., Kenwright A., Schutten M., Boucher C.A., Monto A., Clinch B., Kumar D., Whitley R., Nguyen-Van-Tam J.S. (2019). Viral Kinetics and Resistance Development in Children Treated with Neuraminidase Inhibitors: The Influenza Resistance Information Study (IRIS). Clin. Infect. Dis..

[97] Beigel J.H., Aga E., Elie-Turenne M.C., Cho J., Tebas P., Clark C.L., Metcalf J.P., Ozment C., Raviprakash K., Beeler J. (2019). Anti-influenza immune plasma for the treatment of patients with severe influenza A: A randomised, double-blind, phase 3 trial. Lancet Respir. Med..

[98] Sedeyn K., Saelens X. (2019). New antibody-based prevention and treatment options for influenza. Antivir. Res.

[99] Vermillion M.S., Ursin R.L., Kuok D.I.T., Vom Steeg L.G., Wohlgemuth N., Hall O.J., Fink A.L., Sasse E., Nelson A., Ndeh R. (2018). Production of amphiregulin and recovery from influenza is greater in males than females. Biol. Sex Differ..

[100] Sun J., Madan R., Karp C.L., Braciale T.J. (2009). Effector T cells control lung inflammation during acute influenza virus infection by producing IL-10. Nat. Med..

[101] Tate M.D., Schilter H.C., Brooks A.G., Reading P.C. (2011). Responses of mouse airway epithelial cells and alveolar macrophages to virulent and avirulent strains of influenza A virus. Viral Immunol..

[102] Engeland C.G., Bosch J.A., Cacioppo J.T., Marucha P.T. (2006). Mucosal wound healing: The roles of age and sex. Arch. Surg..

[103] Bandyopadhyay G., Huyck H.L., Misra R.S., Bhattacharya S., Wang Q., Mereness J., Lillis J., Myers J.R., Ashton J., Bushnell T. (2018). Dissociation, cellular isolation, and initial molecular characterization of neonatal and pediatric human lung tissues. Am. J. Physiol. Lung Cell. Mol. Physiol..

[104] Kyle J.E., Clair G., Bandyopadhyay G., Misra R.S., Zink E.M., Bloodsworth K.J., Shukla A.K., Du Y., Lillis J., Myers J.R. (2018). Cell type-resolved human lung lipidome reveals cellular cooperation in lung function. Sci. Rep..

[105] Du Y., Clair G.C., Al Alam D., Danopoulos S., Schnell D., Kitzmiller J.A., Misra R.S., Bhattacharya S., Warburton D., Mariani T.J. (2019). Integration of transcriptomic and proteomic data identifies biological functions in cell populations from human infant lung. Am. J. Physiol. Lung Cell. Mol. Physiol..

[106] Simon A.K., Hollander G.A., McMichael A. (2015). Evolution of the immune system in humans from infancy to old age. Proc. Biol. Sci..

[107] Esteve-Sole A., Luo Y., Vlagea A., Deya-Martinez A., Yague J., Plaza-Martin A.M., Juan M., Alsina L. (2018). B Regulatory Cells: Players in Pregnancy and Early Life. Int. J. Mol. Sci..

[108] Tosato F., Bucciol G., Pantano G., Putti M.C., Sanzari M.C., Basso G., Plebani M. (2015). Lymphocytes subsets reference values in childhood. Cytometry A.

[109] Wang G., Miyahara Y., Guo Z., Khattar M., Stepkowski S.M., Chen W. (2010). “Default” generation of neonatal regulatory T cells. J. Immunol..

[110] Takahata Y., Nomura A., Takada H., Ohga S., Furuno K., Hikino S., Nakayama H., Sakaguchi S., Hara T. (2004). CD25+CD4+ T cells in human cord blood: An immunoregulatory subset with naive phenotype and specific expression of forkhead box p3 (Foxp3) gene. Exp. Hematol..

[111] Sarvaria A., Basar R., Mehta R.S., Shaim H., Muftuoglu M., Khoder A., Sekine T., Gokdemir E., Kondo K., Marin D. (2016). IL-10+ regulatory B cells are enriched in cord blood and may protect against cGVHD after cord blood transplantation. Blood.

[112] Esteve-Sole A., Teixido I., Deya-Martinez A., Yague J., Plaza-Martin A.M., Juan M., Alsina L. (2017). Characterization of the Highly Prevalent Regulatory CD24(hi)CD38(hi) B-Cell Population in Human Cord Blood. Front. Immunol..

[113] Liu P., Li L., Fan P., Zheng J., Zhao D. (2018). High-dose of intravenous immunoglobulin modulates immune tolerance in premature infants. BMC Pediatr..

[114] Rueda C.M., Moreno-Fernandez M.E., Jackson C.M., Kallapur S.G., Jobe A.H., Chougnet C.A. (2015). Neonatal regulatory T cells have reduced capacity to suppress dendritic cell function. Eur. J. Immunol..

[115] Coates B.M., Staricha K.L., Wiese K.M., Ridge K.M. (2015). Influenza A Virus Infection, Innate Immunity, and Childhood. JAMA Pediatr..

[116] Papaioannou N.E., Pasztoi M., Schraml B.U. (2018). Understanding the Functional Properties of Neonatal Dendritic Cells: A Doorway to Enhance Vaccine Effectiveness?. Front. Immunol..

[117] Lawrence S.M., Corriden R., Nizet V. (2017). Age-Appropriate Functions and Dysfunctions of the Neonatal Neutrophil. Front. Pediatr..

[118] Kollmann T.R., Kampmann B., Mazmanian S.K., Marchant A., Levy O. (2017). Protecting the Newborn and Young Infant from Infectious Diseases: Lessons from Immune Ontogeny. Immunity.

[119] Wisgrill L., Groschopf A., Herndl E., Sadeghi K., Spittler A., Berger A., Forster-Waldl E. (2016). Reduced TNF-alpha response in preterm neonates is associated with impaired nonclassic monocyte function. J. Leukoc. Biol..

[120] de Jong E., Strunk T., Burgner D., Lavoie P.M., Currie A. (2017). The phenotype and function of preterm infant monocytes: Implications for susceptibility to infection. J. Leukoc. Biol..

[121] Collins A., Weitkamp J.H., Wynn J.L. (2018). Why are preterm newborns at increased risk of infection? Archives of disease in childhood. Fetal Neonatal Ed..

[122] D’Angio C.T., Wyman C.P., Misra R.S., Halliley J.L., Wang H., Hunn J.E., Fallone C.M., Lee F.E. (2017). Plasma cell and serum antibody responses to influenza vaccine in preterm and full-term infants. Vaccine.

[123] D’Angio C.T., Heyne R.J., Duara S., Holmes L.C., O’Shea T.M., Wang H., Wang D., Sanchez P.J., Welliver R.C., Ryan R.M. (2011). Immunogenicity of trivalent influenza vaccine in extremely low-birth-weight, premature versus term infants. Pediatr. Infect. Dis. J..

[124] Misra R., Shah S., Fowell D., Wang H., Scheible K., Misra S., Huyck H., Wyman C., Ryan R.M., Reynolds A.M. (2015). Preterm cord blood CD4(+) T cells exhibit increased IL-6 production in chorioamnionitis and decreased CD4(+) T cells in bronchopulmonary dysplasia. Hum. Immunol..

[125] Jobe A.H. (2016). Mechanisms of Lung Injury and Bronchopulmonary Dysplasia. Am. J. Perinatol..

[126] Mitra S., Disher T., Pichler G., D’Souza B., McCord H., Chayapathi V., Jones K., Schmolzer G. (2019). Delivery room interventions to prevent bronchopulmonary dysplasia in preterm infants: A protocol for a systematic review and network meta-analysis. BMJ Open.

[127] Coalson J.J. (2006). Pathology of bronchopulmonary dysplasia. Semin. Perinatol..

[128] Papagianis P.C., Pillow J.J., Moss T.J. (2019). Bronchopulmonary dysplasia: Pathophysiology and potential anti-inflammatory therapies. Paediatr. Respir. Rev..

[129] Collins J.J.P., Tibboel D., de Kleer I.M., Reiss I.K.M., Rottier R.J. (2017). The Future of Bronchopulmonary Dysplasia: Emerging Pathophysiological Concepts and Potential New Avenues of Treatment. Front. Med..

[130] Bhandari A., Carroll C., Bhandari V. (2016). BPD Following Preterm Birth: A Model for Chronic Lung Disease and a Substrate for ARDS in Childhood. Front. Pediatr..

[131] Homaira N., Briggs N., Oei J.L., Hilder L., Bajuk B., Snelling T., Chambers G.M., Jaffe A. (2019). Impact of influenza on hospitalization rates in children with a range of chronic lung diseases. Influenza Other Respir. Viruses.

[132] Black S., Nicolay U., Vesikari T., Knuf M., Del Giudice G., Della Cioppa G., Tsai T., Clemens R., Rappuoli R. (2011). Hemagglutination inhibition antibody titers as a correlate of protection for inactivated influenza vaccines in children. Pediatr. Infect. Dis. J..

[133] Wilkinson T.M., Li C.K., Chui C.S., Huang A.K., Perkins M., Liebner J.C., Lambkin-Williams R., Gilbert A., Oxford J., Nicholas B. (2012). Preexisting influenza-specific CD4(+) T cells correlate with disease protection against influenza challenge in humans. Nat. Med..

[134] Sridhar S., Begom S., Bermingham A., Hoschler K., Adamson W., Carman W., Bean T., Barclay W., Deeks J.J., Lalvani A. (2013). Cellular immune correlates of protection against symptomatic pandemic influenza. Nat. Med..

[135] Belshe R.B., Edwards K.M., Vesikari T., Black S.V., Walker R.E., Hultquist M., Kemble G., Connor E.M., Group C.-T.C.E.S. (2007). Live attenuated versus inactivated influenza vaccine in infants and young children. N. Engl. J. Med..

[136] Grohskopf L.A., Alyanak E., Broder K.R., Walter E.B., Fry A.M., Jernigan D.B. (2019). Prevention and Control of Seasonal Influenza with Vaccines: Recommendations of the Advisory Committee on Immunization Practices-United States, 2019–2020 Influenza Season. MMWR Recomm. Rep..

[137] ACOG Committee on Obstetric Practice (2018). ACOG Committee Opinion No. 732: Influenza Vaccination During Pregnancy. Obstet. Gynecol..

[138] Gagneur A., Pinquier D., Quach C. (2015). Immunization of preterm infants. Hum. Vaccines Immunother..

[139] Center for Disease Control and Prevention Special Situations; General Best Practice Guidelines for Immunization: Best Practices Guidance of the Advisory Committee on Immunization Practices (ACIP). https://www.cdc.gov/vaccines/hcp/acip-recs/general-recs/special-situations.html.

[140] Ortiz J.R., Perut M., Dumolard L., Wijesinghe P.R., Jorgensen P., Ropero A.M., Danovaro-Holliday M.C., Heffelfinger J.D., Tevi-Benissan C., Teleb N.A. (2016). A global review of national influenza immunization policies: Analysis of the 2014 WHO/UNICEF Joint Reporting Form on immunization. Vaccine.

[141] Paules C., Subbarao K. (2017). Influenza. Lancet.

[142] Rajao D.S., Gauger P.C., Anderson T.K., Lewis N.S., Abente E.J., Killian M.L., Perez D.R., Sutton T.C., Zhang J., Vincent A.L. (2015). Novel Reassortant Human-Like H3N2 and H3N1 Influenza A Viruses Detected in Pigs Are Virulent and Antigenically Distinct from Swine Viruses Endemic to the United States. J. Virol..

[143] Zhang W., Hirve S., Kieny M.P. (2017). Seasonal vaccines—Critical path to pandemic influenza response. Vaccine.

[144] Sheikh S., Biundo E., Courcier S., Damm O., Launay O., Maes E., Marcos C., Matthews S., Meijer C., Poscia A. (2018). A report on the status of vaccination in Europe. Vaccine.

[145] Ohlrogge A.W., Suggs L.S. (2018). Flu vaccination communication in Europe: What does the government communicate and how?. Vaccine.

[146] Samaan G., McPherson M., Partridge J. (2013). A review of the evidence to support influenza vaccine introduction in countries and areas of WHO’s Western Pacific Region. PLoS ONE.

[147] Fischer W.A., Gong M., Bhagwanjee S., Sevransky J. (2014). Global burden of influenza as a cause of cardiopulmonary morbidity and mortality. Glob. Heart.

[148] Lu P.J., Hung M.C., O’Halloran A.C., Ding H., Srivastav A., Williams W.W., Singleton J.A. (2019). Seasonal Influenza Vaccination Coverage Trends Among Adult Populations, U.S., 2010–2016. Am. J. Prev. Med..

[149] Buchan S.A., Kwong J.C. (2016). Trends in influenza vaccine coverage and vaccine hesitancy in Canada, 2006/07 to 2013/14: Results from cross-sectional survey data. CMAJ Open.

[150] Ding H., Kahn K.E., Black C.L., O’Halloran A., Lu P.J., Williams W.W. (2019). Influenza Vaccination Coverage Among Pregnant Women in the U.S., 2012–2015. Am. J. Prev. Med..

[151] Flannery B., Reynolds S.B., Blanton L., Santibanez T.A., O’Halloran A., Lu P.-J., Chen J., Foppa I.M., Gargiullo P., Bresee J. (2017). Influenza Vaccine Effectiveness Against Pediatric Deaths: 2010–2014. Pediatrics.

[152] Hartel C., Humberg A., Viemann D., Stein A., Orlikowsky T., Rupp J., Kopp M.V., Herting E., Gopel W. (2016). Preterm Birth during Influenza Season Is Associated with Adverse Outcome in Very Low Birth Weight Infants. Front. Pediatrics.

[153] Neuzil K.M., Mellen B.G., Wright P.F., Mitchel E.F., Griffin M.R. (2000). The effect of influenza on hospitalizations, outpatient visits, and courses of antibiotics in children. N. Engl. J. Med..

[154] Hofstetter A.M., Jacobson E.N., deHart M.P., Englund J.A. (2019). Early Childhood Vaccination Status of Preterm Infants. Pediatrics.

[155] World Health Organization (2012). Vaccines against influenza WHO position paper—November 2012. Wkly. Epidemiol. Rec..

[156] Mertz D., Lo C.K., Lytvyn L., Ortiz J.R., Loeb M., Flurisk I. (2019). Pregnancy as a risk factor for severe influenza infection: An individual participant data meta-analysis. BMC Infect. Dis..

[157] Mertz D., Kim T.H., Johnstone J., Lam P.P., Science M., Kuster S.P., Fadel S.A., Tran D., Fernandez E., Bhatnagar N. (2013). Populations at risk for severe or complicated influenza illness: Systematic review and meta-analysis. BMJ.

[158] Mertz D., Geraci J., Winkup J., Gessner B.D., Ortiz J.R., Loeb M. (2017). Pregnancy as a risk factor for severe outcomes from influenza virus infection: A systematic review and meta-analysis of observational studies. Vaccine.

[159] Sperling R.S., Riley L.E., Immunization and Emerging Infections Expert Work Group (2018). Influenza Vaccination, Pregnancy Safety, and Risk of Early Pregnancy Loss. Obstet. Gynecol..

[160] Zerbo O., Modaressi S., Chan B., Goddard K., Lewis N., Bok K., Fireman B., Klein N.P., Baxter R. (2017). No association between influenza vaccination during pregnancy and adverse birth outcomes. Vaccine.

[161] Walsh L.K., Donelle J., Dodds L., Hawken S., Wilson K., Benchimol E.I., Chakraborty P., Guttmann A., Kwong J.C., MacDonald N.E. (2019). Health outcomes of young children born to mothers who received 2009 pandemic H1N1 influenza vaccination during pregnancy: Retrospective cohort study. BMJ.

[162] McHugh L., Marshall H.S., Perrett K.P., Nolan T., Wood N., Lambert S.B., Richmond P., Ware R.S., Binks P., Binks M.J. (2019). The Safety of Influenza and Pertussis Vaccination in Pregnancy in a Cohort of Australian Mother-Infant Pairs, 2012–2015: FluMum Study. Clin. Infect. Dis..

[163] Chambers C.D., Johnson D.L., Xu R., Luo Y.J., Louik C., Mitchell A.A., Schatz M., Jones K.L., OTIS Collaborative Research Group (2016). Safety of the 2010-11, 2011–2012, 2012–2013, and 2013-14 seasonal influenza vaccines in pregnancy: Birth defects, spontaneous abortion, preterm delivery, and small for gestational age infants, a study from the cohort arm of VAMPSS. Vaccine.

[164] Moro P., Baumblatt J., Lewis P., Cragan J., Tepper N., Cano M. (2017). Surveillance of Adverse Events After Seasonal Influenza Vaccination in Pregnant Women and Their Infants in the Vaccine Adverse Event Reporting System, July 2010–May 2016. Drug Saf..

[165] Eaton A., Lewis N., Fireman B., Hansen J., Baxter R., Gee J., Klein N.P. (2018). Birth outcomes following immunization of pregnant women with pandemic H1N1 influenza vaccine 2009–2010. Vaccine.

[166] Nunes M.C., Aqil A.R., Omer S.B., Madhi S.A. (2016). The Effects of Influenza Vaccination during Pregnancy on Birth Outcomes: A Systematic Review and Meta-Analysis. Am. J. Perinatol..

[167] Hutcheon J.A., Fell D.B., Jackson M.L., Kramer M.S., Ortiz J.R., Savitz D.A., Platt R.W. (2016). Detectable Risks in Studies of the Fetal Benefits of Maternal Influenza Vaccination. Am. J. Epidemiol..

[168] Zhang C., Wang X., Liu D., Zhang L., Sun X. (2018). A systematic review and meta-analysis of fetal outcomes following the administration of influenza A/H1N1 vaccination during pregnancy. Int. J. Gynaecol. Obstet..

[169] Kallen B., Olausson P.O. (2012). Vaccination against H1N1 influenza with Pandemrix((R)) during pregnancy and delivery outcome: A Swedish register study. BJOG.

[170] van der Maas N., Dijs-Elsinga J., Kemmeren J., van Lier A., Knol M., de Melker H. (2016). Safety of vaccination against influenza A (H1N1) during pregnancy in the Netherlands: Results on pregnancy outcomes and infant’s health: Cross-sectional linkage study. BJOG.

[171] He J., Liu Z.W., Lu Y.P., Li T.Y., Liang X.J., Arck P.C., Huang S.M., Hocher B., Chen Y.P. (2017). A Systematic Review and Meta-Analysis of Influenza A Virus Infection During Pregnancy Associated with an Increased Risk for Stillbirth and Low Birth Weight. Kidney Blood Press. Res..

[172] Ribeiro A.F., Pellini A.C.G., Kitagawa B.Y., Marques D., Madalosso G., Fred J., Albernaz R.K.M., Carvalhanas T., Zanetta D.M.T. (2018). Severe influenza A(H1N1)pdm09 in pregnant women and neonatal outcomes, State of Sao Paulo, Brazil, 2009. PLoS ONE.

[173] Newsome K., Alverson C.J., Williams J., McIntyre A.F., Fine A.D., Wasserman C., Lofy K.H., Acosta M., Louie J.K., Jones-Vessey K. (2019). Outcomes of infants born to women with influenza A(H1N1)pdm09. Birth Defects Res..

[174] Fell D.B., Savitz D.A., Kramer M.S., Gessner B.D., Katz M.A., Knight M., Luteijn J.M., Marshall H., Bhat N., Gravett M.G. (2017). Maternal influenza and birth outcomes: Systematic review of comparative studies. BJOG.

[175] Madhi S.A., Cutland C.L., Kuwanda L., Weinberg A., Hugo A., Jones S., Adrian P.V., Niekerk N.V., Treurnicht F., Ortiz J.R. (2014). Influenza Vaccination of Pregnant Women and Protection of Their Infants. N. Engl. J. Med..

[176] Nunes M.C., Cutland C.L., Dighero B., Bate J., Jones S., Hugo A., van Niekerk N., Kuwanda L., Izu A., Weinberg A. (2015). Kinetics of Hemagglutination-Inhibiting Antibodies Following Maternal Influenza Vaccination Among Mothers With and Those Without HIV Infection and Their Infants. J. Infect. Dis..

[177] Malek A., Sager R., Kuhn P., Nicolaides K.H., Schneider H. (1996). Evolution of maternofetal transport of immunoglobulins during human pregnancy. Am. J. Reprod. Immunol..

[178] Palmeira P., Costa-Carvalho B.T., Arslanian C., Pontes G.N., Nagao A.T., Carneiro-Sampaio M.M. (2009). Transfer of antibodies across the placenta and in breast milk from mothers on intravenous immunoglobulin. Pediatric Allergy Immunol..

[179] Nunes M.C., Cutland C.L., Jones S., Downs S., Weinberg A., Ortiz J.R., Neuzil K.M., Simões E.A.F., Klugman K.P., Madhi S.A. (2017). Efficacy of Maternal Influenza Vaccination Against All-Cause Lower Respiratory Tract Infection Hospitalizations in Young Infants: Results From a Randomized Controlled Trial. Clin. Infect. Dis..

[180] Zaman K., Roy E., Arifeen S.E., Rahman M., Raqib R., Wilson E., Omer S.B., Shahid N.S., Breiman R.F., Breiman R.E. (2008). Effectiveness of Maternal Influenza Immunization in Mothers and Infants. N. Engl. J. Med..

[181] Benowitz I., Esposito D.B., Gracey K.D., Shapiro E.D., Vázquez M. (2010). Influenza Vaccine Given to Pregnant Women Reduces Hospitalization Due to Influenza in Their Infants. Clin. Infect. Dis..

[182] Zhong Z., Haltalli M., Holder B., Rice T., Donaldson B., O’Driscoll M., Le-Doare K., Kampmann B., Tregoning J.S. (2019). The impact of timing of maternal influenza immunization on infant antibody levels at birth. Clin. Exp. Immunol..

[183] Schlaudecker E.P., Steinhoff M.C., Omer S.B., McNeal M.M., Roy E., Arifeen S.E., Dodd C.N., Raqib R., Breiman R.F., Zaman K. (2013). IgA and neutralizing antibodies to influenza a virus in human milk: A randomized trial of antenatal influenza immunization. PLoS ONE.

[184] Jarvinen K.M., Wang J., Seppo A.E., Zand M. (2018). Novel multiplex assay for profiling influenza antibodies in breast milk and serum of mother-infant pairs. F1000Research.

[185] Brady R.C., Jackson L.A., Frey S.E., Shane A.L., Walter E.B., Swamy G.K., Schlaudecker E.P., Szefer E., Wolff M., McNeal M.M. (2018). Randomized trial comparing the safety and antibody responses to live attenuated versus inactivated influenza vaccine when administered to breastfeeding women. Vaccine.

[186] Demers-Mathieu V., Underwood M.A., Beverly R.L., Nielsen S.D., Dallas D.C. (2018). Comparison of Human Milk Immunoglobulin Survival during Gastric Digestion between Preterm and Term Infants. Nutrients.

[187] van den Berg J.P., Westerbeek E.A., Berbers G.A., van Gageldonk P.G., van der Klis F.R., van Elburg R.M. (2010). Transplacental transport of IgG antibodies specific for pertussis, diphtheria, tetanus, haemophilus influenzae type b, and Neisseria meningitidis serogroup C is lower in preterm compared with term infants. Pediatric Infect. Dis. J..

[188] Demers-Mathieu V., Huston R.K., Markell A.M., McCulley E.A., Martin R.L., Dallas D.C. (2019). Antenatal Influenza A-Specific IgA, IgM, and IgG Antibodies in Mother’s Own Breast Milk and Donor Breast Milk, and Gastric Contents and Stools from Preterm Infants. Nutrients.

[189] Sunwoo S.Y., Schotsaert M., Morozov I., Davis A.S., Li Y., Lee J., McDowell C., Meade P., Nachbagauer R., Garcia-Sastre A. (2018). A Universal Influenza Virus Vaccine Candidate Tested in a Pig Vaccination-Infection Model in the Presence of Maternal Antibodies. Vaccines.

[190] Grabarics M., Csernak O., Balogh R., Beni S. (2017). Analytical characterization of human milk oligosaccharides-potential applications in pharmaceutical analysis. J. Pharm. Biomed. Anal..

[191] Trend S., Strunk T., Lloyd M.L., Kok C.H., Metcalfe J., Geddes D.T., Lai C.T., Richmond P., Doherty D.A., Simmer K. (2016). Levels of innate immune factors in preterm and term mothers’ breast milk during the 1st month postpartum. Br. J. Nutr..

[192] Bedin A.S., Moles J.P., Rutagwera D., Nagot N., Kankasa C., Tylleskar T., Valverde-Villegas J.M., Durand M., Van de Perre P., Tuaillon E. (2019). MAIT cells, TCR gammadelta+ cells and ILCs cells in human breast milk and blood from HIV infected and uninfected women. Pediatric Allergy Immunol..

[193] Xiao L., Leusink-Muis T., Kettelarij N., van Ark I., Blijenberg B., Hesen N.A., Stahl B., Overbeek S.A., Garssen J., Folkerts G. (2018). Human Milk Oligosaccharide 2′-Fucosyllactose Improves Innate and Adaptive Immunity in an Influenza-Specific Murine Vaccination Model. Front. Immunol..

[194] Coulet M., Phothirath P., Allais L., Schilter B. (2014). Pre-clinical safety evaluation of the synthetic human milk, nature-identical, oligosaccharide 2′-O-Fucosyllactose (2′FL). Regul. Toxicol. Pharmacol..

[195] Vazquez E., Barranco A., Ramirez M., Gruart A., Delgado-Garcia J.M., Martinez-Lara E., Blanco S., Martin M.J., Castanys E., Buck R. (2015). Effects of a human milk oligosaccharide, 2′-fucosyllactose, on hippocampal long-term potentiation and learning capabilities in rodents. J. Nutr. Biochem..

[196] Elison E., Vigsnaes L.K., Rindom Krogsgaard L., Rasmussen J., Sorensen N., McConnell B., Hennet T., Sommer M.O., Bytzer P. (2016). Oral supplementation of healthy adults with 2′-O-fucosyllactose and lacto-N-neotetraose is well tolerated and shifts the intestinal microbiota. Br. J. Nutr..

[197] Tregoning J.S., Russell R.F., Kinnear E. (2018). Adjuvanted influenza vaccines. Hum. Vaccines Immunother..

[198] Diez-Domingo J., Garces-Sanchez M., Baldo J.M., Planelles M.V., Ubeda I., JuBert A., Mares J., Moris P., Garcia-Corbeira P., Drame M. (2010). Immunogenicity and Safety of H5N1 A/Vietnam/1194/2004 (Clade 1) AS03-adjuvanted prepandemic candidate influenza vaccines in children aged 3 to 9 years: A phase ii, randomized, open, controlled study. Pediatric Infect. Dis. J..

[199] Waddington C.S., Walker W.T., Oeser C., Reiner A., John T., Wilkins S., Casey M., Eccleston P.E., Allen R.J., Okike I. (2010). Safety and immunogenicity of AS03B adjuvanted split virion versus non-adjuvanted whole virion H1N1 influenza vaccine in UK children aged 6 months-12 years: Open label, randomised, parallel group, multicentre study. BMJ.

[200] Monto A.S., Malosh R.E., Petrie J.G., Martin E.T. (2017). The Doctrine of Original Antigenic Sin: Separating Good From Evil. J. Infect. Dis..

[201] Jackson L.A., Chen W.H., Stapleton J.T., Dekker C.L., Wald A., Brady R.C., Edupuganti S., Winokur P., Mulligan M.J., Keyserling H.L. (2012). Immunogenicity and safety of varying dosages of a monovalent 2009 H1N1 influenza vaccine given with and without AS03 adjuvant system in healthy adults and older persons. J. Infect. Dis..

[202] Carmona A., Omenaca F., Tejedor J.C., Merino J.M., Vaman T., Dieussaert I., Gillard P., Aristegui J. (2010). Immunogenicity and safety of AS03-adjuvanted 2009 influenza A H1N1 vaccine in children 6–35 months. Vaccine.

[203] Garcia-Sicilia J., Gillard P., Carmona A., Tejedor J.C., Aristegui J., Merino J.M., Behre U., Caplanusi A., Vaman T., Dieussaert I. (2011). Immunogenicity and safety of AS03-adjuvanted H1N1 pandemic vaccines in children and adolescents. Vaccine.

[204] Laupeze B., Herve C., Di Pasquale A., Tavares Da Silva F. (2019). Adjuvant Systems for vaccines: 13years of post-licensure experience in diverse populations have progressed the way adjuvanted vaccine safety is investigated and understood. Vaccine.

[205] Esposito S., Pugni L., Daleno C., Ronchi A., Valzano A., Serra D., Mosca F., Principi N. (2011). Influenza A/H1N1 MF59-adjuvanted vaccine in preterm and term children aged 6 to 23 months. Pediatrics.

[206] Nakaya H.I., Clutterbuck E., Kazmin D., Wang L., Cortese M., Bosinger S.E., Patel N.B., Zak D.E., Aderem A., Dong T. (2016). Systems biology of immunity to MF59-adjuvanted versus nonadjuvanted trivalent seasonal influenza vaccines in early childhood. Proc. Natl. Acad. Sci. USA.

[207] Zedda L., Forleo-Neto E., Vertruyen A., Raes M., Marchant A., Jansen W., Clouting H., Arora A., Beatty M.E., Galli G. (2015). Dissecting the immune response to MF59-adjuvanted and nonadjuvanted seasonal influenza vaccines in children less than three years of age. Pediatr. Infect. Dis. J..

[208] Mastelic Gavillet B., Eberhardt C.S., Auderset F., Castellino F., Seubert A., Tregoning J.S., Lambert P.H., de Gregorio E., Del Giudice G., Siegrist C.A. (2015). MF59 Mediates Its B Cell Adjuvanticity by Promoting T Follicular Helper Cells and Thus Germinal Center Responses in Adult and Early Life. J. Immunol..

[209] Haber P., Moro P.L., Ng C., Dores G.M., Lewis P., Cano M. (2019). Post-licensure surveillance of trivalent adjuvanted influenza vaccine (aIIV3; Fluad), Vaccine Adverse Event Reporting System (VAERS), United States, July 2016–June 2018. Vaccine.

[210] Sakala I.G., Honda-Okubo Y., Fung J., Petrovsky N. (2016). Influenza immunization during pregnancy: Benefits for mother and infant. Hum. Vaccines Immunother..

[211] Baum U., Leino T., Gissler M., Kilpi T., Jokinen J. (2015). Perinatal survival and health after maternal influenza A(H1N1)pdm09 vaccination: A cohort study of pregnancies stratified by trimester of vaccination. Vaccine.

[212] Ludvigsson J.F., Strom P., Lundholm C., Cnattingius S., Ekbom A., Ortqvist A., Feltelius N., Granath F., Stephansson O. (2015). Maternal vaccination against H1N1 influenza and offspring mortality: Population based cohort study and sibling design. BMJ.

[213] Miller E., Andrews N., Stellitano L., Stowe J., Winstone A.M., Shneerson J., Verity C. (2013). Risk of narcolepsy in children and young people receiving AS03 adjuvanted pandemic A/H1N1 2009 influenza vaccine: Retrospective analysis. BMJ Br. Med. J..

[214] Sarkanen T.O., Alakuijala A.P.E., Dauvilliers Y.A., Partinen M.M. (2018). Incidence of narcolepsy after H1N1 influenza and vaccinations: Systematic review and meta-analysis. Sleep Med. Rev..

[215] Verstraeten T., Cohet C., Santos G.D., Ferreira G.L.C., Bollaerts K., Bauchau V., Shinde V. (2015). Pandemrix™ and narcolepsy: A critical appraisal of the observational studies. Hum. Vaccines Immunother..

[216] McClenathan B.M., Edwards K.M. (2019). Vaccine safety: An evolving evidence-based science. Br. J. Clin. Pharmacol..

[217] Kaczmarek M.C., Duong U.T., Ware R.S., Lambert S.B., Kelly H.A. (2013). The risk of fever following one dose of trivalent inactivated influenza vaccine in children aged >/=6 months to <36 months: A comparison of published and unpublished studies. Vaccine.

[218] Halsey N.A., Talaat K.R., Greenbaum A., Mensah E., Dudley M.Z., Proveaux T., Salmon D.A. (2015). The safety of influenza vaccines in children: An Institute for Vaccine Safety white paper. Vaccine.

[219] Stone C.A., Rukasin C.R.F., Beachkofsky T.M., Phillips E.J. (2019). Immune Mediated Adverse Reactions to Vaccines. Br. J. Clin. Pharmacol..

[220] Iaconelli J., Xuan L., Karmacharya R. (2019). HDAC6 Modulates Signaling Pathways Relevant to Synaptic Biology and Neuronal Differentiation in Human Stem-Cell-Derived Neurons. Int. J. Mol. Sci..

[221] Shimabukuro T.T., Nguyen M., Martin D., DeStefano F. (2015). Safety monitoring in the Vaccine Adverse Event Reporting System (VAERS). Vaccine.

[222] Rizzo C., Rezza G., Ricciardi W. (2018). Strategies in recommending influenza vaccination in Europe and US. Hum. Vaccines Immunother..

[223] O’Leary S.T., Riley L.E., Lindley M.C., Allison M.A., Albert A.P., Fisher A., Jiles A.J., Crane L.A., Hurley L.P., Beaty B. (2019). Obstetrician-Gynecologists’ Strategies to Address Vaccine Refusal Among Pregnant Women. Obstet. Gynecol..

[224] Donzelli A. (2018). Influenza Vaccinations for All Pregnant Women? Better Evidence Is Needed. Int. J. Environ. Res. Public Health.

[225] Bresee J.S., Lafond K.E., McCarron M., Azziz-Baumgartner E., Chu S.Y., Ebama M., Hinman A.R., Xeuatvongsa A., Bino S., Richardson D. (2019). The partnership for influenza vaccine introduction (PIVI): Supporting influenza vaccine program development in low and middle-income countries through public-private partnerships. Vaccine.

[226] Ellingson M.K., Dudley M.Z., Limaye R.J., Salmon D.A., O’Leary S.T., Omer S.B. (2019). Enhancing uptake of influenza maternal vaccine. Expert Rev. Vaccines.

[227] Halder N., Kelso J.K., Milne G.J. (2014). A model-based economic analysis of pre-pandemic influenza vaccination cost-effectiveness. BMC Infect. Dis..

[228] Metcalf C.J.E., Ferrari M., Graham A.L., Grenfell B.T. (2015). Understanding Herd Immunity. Trends Immunol..

[229] Eichner M., Schwehm M., Eichner L., Gerlier L. (2017). Direct and indirect effects of influenza vaccination. BMC Infect. Dis..

[230] Switzer C., Babiuk L., Loeb M. (2019). Determining optimal community protection strategies for the influenza vaccine. Expert Rev. Vaccines.

[231] National Insititue of Health and Care Excellence Evidence Reviews for Increasing Uptake in Children. https://www.nice.org.uk/guidance/ng103/resources/flu-vaccination-increasing-uptake-pdf-66141536272837.

[232] Betsch C., Bohm R. (2016). Detrimental effects of introducing partial compulsory vaccination: Experimental evidence. Eur. J. Public Health.

[233] Omer S.B., Betsch C., Leask J. (2019). Mandate vaccination with care. Nature.

[234] Poland G.A. (2011). MMR vaccine and autism: Vaccine nihilism and postmodern science. Mayo Clin. Proc..

[235] (2010). Retraction-Ileal-lymphoid-nodular hyperplasia, non-specific colitis, and pervasive developmental disorder in children. Lancet.

[236] Taylor L.E., Swerdfeger A.L., Eslick G.D. (2014). Vaccines are not associated with autism: An evidence-based meta-analysis of case-control and cohort studies. Vaccine.

[237] Zerbo O., Modaressi S., Goddard K., Lewis E., Fireman B.H., Daley M.F., Irving S.A., Jackson L.A., Donahue J.G., Qian L. (2018). Vaccination Patterns in Children After Autism Spectrum Disorder Diagnosis and in Their Younger Siblings. JAMA Pediatr..

[238] Vanderslott S. (2019). Study shows lower vaccination rates for younger siblings after autism spectrum disorder diagnosis in older siblings. Evid. Based Nurs..

[239] Lane S., MacDonald N.E., Marti M., Dumolard L. (2018). Vaccine hesitancy around the globe: Analysis of three years of WHO/UNICEF Joint Reporting Form data-2015–2017. Vaccine.

[240] Nihlen Fahlquist J. (2018). Vaccine hesitancy and trust. Ethical aspects of risk communication. Scand. J. Public Health.

[241] Debellut F., Hendrix N., Ortiz J.R., Lambach P., Neuzil K.M., Bhat N., Pecenka C. (2018). Forecasting demand for maternal influenza immunization in low- and lower-middle-income countries. PLoS ONE.

[242] Launay O., Tsatsaris V. (2016). Maternal influenza immunisation in resource-limited settings. Lancet Infect. Dis..

[243] Tapia M.D., Sow S.O., Tamboura B., Teguete I., Pasetti M.F., Kodio M., Onwuchekwa U., Tennant S.M., Blackwelder W.C., Coulibaly F. (2016). Maternal immunisation with trivalent inactivated influenza vaccine for prevention of influenza in infants in Mali: A prospective, active-controlled, observer-blind, randomised phase 4 trial. Lancet Infect. Dis..

[244] Breteler J.K., Tam J.S., Jit M., Ket J.C., De Boer M.R. (2013). Efficacy and effectiveness of seasonal and pandemic A (H1N1) 2009 influenza vaccines in low and middle income countries: A systematic review and meta-analysis. Vaccine.

[245] Ortiz J.R., Neuzil K.M. (2017). Influenza immunization of pregnant women in resource-constrained countries: An update for funding and implementation decisions. Curr. Opin. Infect. Dis..

[246] Global_Alliance_for_Vaccines_and_Immunisation_Alliance Vaccine Investment Strategy. https://www.gavi.org/about/strategy/vaccine-investment-strategy/.

[247] Grijalva C.G., Zhu Y., Williams D.J., Self W.H., Ampofo K., Pavia A.T., Stockmann C.R., McCullers J., Arnold S.R., Wunderink R.G. (2015). Association Between Hospitalization With Community-Acquired Laboratory-Confirmed Influenza Pneumonia and Prior Receipt of Influenza Vaccination. JAMA.

[248] Ortiz J.R., Neuzil K.M., Ahonkhai V.I., Gellin B.G., Salisbury D.M., Read J.S., Adegbola R.A., Abramson J.S. (2012). Translating vaccine policy into action: A report from the Bill & Melinda Gates Foundation Consultation on the prevention of maternal and early infant influenza in resource-limited settings. Vaccine.

